# Hospital and Institutional News

**Published:** 1915-08-28

**Authors:** 


### HOSPITAL AND INSTITUTIONAL NEWS.

### DEATH OF PROFESSOR EHRLICH.

The medical world will learn with much regret of the death of Professor Ehrlich. It is announced that he died suddenly in his laboratory at Homburg on August 20. A native of Silesia, and the son of Jewish parents, he was born in 1854, and graduated in medicine at the University of Strasburg. Everyone will recognise that he has written his name deeply in the medicine of our time, and there are many indications that much of his work will be enduring. To pathologists and physicians he has long been known as an original, courageous, and determined worker and investigator, but to the general public it is the introduction of salvarsan, or "606," which has fixed his fame. As with parallel discoverers in the therapeutic area, the virtues of this substance were at first put on too high a plane, but that it was a large and valuable advance cannot be contested. The most impressive character of the achievement, apart from its utility, was the ordered scheme of investigation which led to its production. Such a note, indeed, was the feature of all Ehrlich's work. With a given problem in front of him he organised a considered plan of campaign for its solution, and until this was accomplished he spared neither time nor pains. No national antagonisms will be allowed—at least in this country—to hinder our gratitude to Ehrlich or to detract from his greatness. At the International Medical Congress held in London two years ago he was selected, with the general approval of the medical profession, to give one of the principal addresses, and he more than justified the compliment paid to him. We pretend to no knowledge of his personal opinions, and we recognise the appeal which patriotism would make to him, but we are sure that he would regret the severance of associations with a country in which he had many friends, and where his high standard of scientific veracity and his devotion to scientific work were widely appreciated.

### MEDICAL SPECIALISM AT THE FRONT.

If scientific knowledge has been applied, as is only too evidently the case, to render war more destructive and more terrible, something, fortunately, may be placed on the other side of the account. What medicine and surgery and hygiene and sanitation have done for our soldiers in Flanders and in the Near East can hardly be stated in a paragraph, but it is certain that bacilli are no longer more deadly than bullets; or, more accurately, their deadliness is nullified by scientific resourcefulness. An army in the field, and practically free from epidemic disease, is the outcome of much ingenious and diligent work in the laboratory. It is because of a stern resolve to get at the actual causes and conditions of epidemics that our armies to-day are not decimated by disease. Concentrated effort on limited and definite problems is now justifying itself in experience. There is another side to the division of medical duties in relation to the fighting at the Front, and it makes pleasant reading. Mr. James O'Grady, M.P. for East Leeds, and a member of the Labour Party, has just returned from France and Flanders, and he gives us an interesting picture of medical work behind the fighting line. All the casualties of war, fortunately, are not of the serious order, and these lesser accidents and injuries, as well as the minor ills to which flesh is heir, are provided for in special hospital arrangements. Serious wounds of various kinds, and peculiar disturbances of the nervous system, no doubt engage the principal attention of the experts who have been sent out from home, but trench fighting does not exclude the possibility of toothache. The dental surgeons, as may be gathered from a recent newspaper correspondence, think the War Office arrangements are open to serious criticism, but according to Mr. O'Grady, "Tommy Atkins" can readily be relieved of an aching molar, or can even get artificial grinders if his own set falls below efficiency. Then provision is made for the treatment of sore feet, and the minor misery of corns—minor, that is, to those who are free from these callosities—produces the sympathetic aid of the skilled chiropodist. Last of all, body and clothes and rifle can all be disinfected, and in this delicious corporeal state the happy warrior is regaled with literature and music. It is a cheering sketch, and though very far from the complete picture, it helps to redress the monotonous and gloomy outlines which some of our stay-at-home prophets so sedulously cultivate.

### A GREAT CANADIAN HOSPITAL AT ORPINGTON.

The Ontario Government has purchased a site near the main South-Eastern line at Orpington for the erection thereon of a Canadian hospital to contain 1,040 beds, to be used primarily for acute cases among the wounded men of the Canadian contingent. Whenever there may be room for convalescents and those suffering from shock they will also be treated at this hospital as far as circumstances permit. The erection of this Canadian hospital has been begun, and is being proceeded with rapidly. The buildings are substantial, and the cost of construction and equipment will be borne by the Ontario Government. It will, we hope, receive a generous grant from the War Office, which is aiding hospitals placed on its recognised voluntary aid list. The great war is proving a mammoth wonder-worker. This important Canadian hospital, erected in England and paid for by Canada, is one of many great facts, having momentous results ahead, possibly.

### MR. IAN MALCOLM, M P.

The appreciation of personal service under the Red Cross at the seat of war has found generous expression by his constituents in the gift to Mr. Ian Malcolm, M.P., one of the kindest and most popular of men, of two motor ambulances which have been presented to Mr. Malcolm, and by him handed over for the use of the War Office to Surgeon-General Russell, who attended with that object on behalf of the Secretary of State.

### NEW CONVALESCENT HOMES.

Lord Decies has offered Beresford Lodge, near Margate, to the committee of the Services' Club as a convalescent home for invalided officers of the British and Allied Forces. It is hoped to make such arrangements that each officer may have a relative or friend with him during convalescence. Dollis Hill House, which is so closely associated with the name of Mr. Gladstone, has been offered by the Earl of Aberdeen as a convalescent home for wounded soldiers. The immediate neighbourhood of the park called after the celebrated statesman obviously adds to the attractions of the new convalescent home.

### DR. ROBERT SINCLAIR, OF DUNDEE.

Among the trusted physicians of Dundee the name of Dr. Sinclair occupies an honoured place. He has been in practice there for forty years, and has gained the respect both of his colleagues and of his fellow-citizens. He has now determined to enjoy a well-earned rest, and the occasion has been taken advantage of to express the regard and affection of those who know him. Many generous regrets at his departure have been recorded, and he has been presented with a silver tray and a cheque for £1,000. Dr. Sinclair is a graduate of the University of Glasgow, and in addition to many civic and professional activities he has been prominently concerned in the organisation of the medical school which has now gained a secure footing in the city of his adoption.

### OFFENDERS IN HIGH PLACES.

To hear of a colonel and a corporal being summoned and fined for failing to obscure the lights in a building used as a military hospital is a somewhat startling experience. Yet this is exactly what recently happened in the good city of Newcastle-on-Tyne. In vain did the colonel urge that the engineers had not yet been able to provide the necessary blinds, and that as, on the evening in question, a convoy of wounded had arrived, it was imperative to have lights in the building. Rules and regulations endorsed by military authority could be quoted against him, and so the "engineer was hoist with his own petard." The chairman of the magistrates remarked that the greatest offenders in the city were those who ought to set a good example, and so, having secured the corporal and the colonel, he utilised them *pour encourager les autres*.

### THE DIFFICULTIES OF PERSONAL IDENTIFICATION.

Medico-legal experts and police officials are not unfamiliar with the confidence with which witnesses will swear to an identification which subsequent events prove to be utterly mistaken. Occasionally even expert observers make similar mistakes, as was shown in a painful and notorious case some few weeks ago. That there may be difficulties in identification when a long interval of time has passed, or when a dead body is altered by putrefactive change, is easy to understand, though the confidence with which it is effected is sometimes amazing. But that a woman should receive a man as her husband and should live with him for some days in this capacity, and should then discover that she doubted whether he was the genuine spouse, is truly surprising. Truth, especially the truth of the Law Courts, is indeed often stranger than fiction.

### RED CROSS WORK IN ABERDEENSHIRE.

The annual report of the county of Aberdeen Branch of the British Red Cross Society is a record of energetic and enterprising work. A number of hospitals for the reception of soldiers have been organised throughout the county, and they have been supplemented by a series of convalescent homes; indeed, the offers of provision in this latter direction have been in excess of the requirements of the position. A second department of activity has been the arrangement of working parties to prepare and supply dressings and other materials for hospitals, and in this direction much energy has been shown. The gross sewn and knitted articles contributed by the county (including the city of Aberdeen) is 36,807. Classes in first-aid and nursing have received a great influx of pupils, and have proved powerful recruiting agencies for the Volunteer Aid Detachments. In the earlier part of the year four fully-equipped motor ambulances were sent to the Front at a cost of £1,755. The president of the branch is the Countess of Aberdeen, and the honorary secretary and treasurer is Mr. William Smith.

### GIFTS AND SERVICE IN WAR-TIME.

Every day produces evidence of the spontaneous willingness and never-ceasing generosity of all classes of the people throughout the Empire. The faith which the authorities have in the unfailing supply of gifts directly a new need is discovered, and the want made public, is proved by the communications they make to the newspapers. Serbia and other distant centres of conflict have had much spontaneous help, and especially from Great Britain. One of the latest demands comes from the Dardanelles, where Sir Courtauld Thomson, commissioner in the Near East for the Joint Committee of the British Red Cross Society and the St. John Ambulance Association, telegraphed for a picket boat to tow barges conveying wounded men from Gallipoli to Mudros, and for two motor launches in addition to carry men and stores. Promptly Mr. John Masefield, the poet and novelist, found the money for the picket and also for a barge, and has proceeded to Mudros that he may take charge of them as soon as they arrive. A twin-screw petrol boat, 32 horse-power, for the picket above referred to, and two motor launches, 20 and 25 horse-power, have been already secured by the Joint Committee. In addition to these, two motor launches have been found for the Persian

Gulf expedition, at the request of Sir John Nixon, and the first has already been dispatched. The need for gifts of further motor boats for the Eastern war field is insisted upon, and we hope some, at any rate, of our readers may send a contribution towards their cost, addressed to 83 Pall Mall, S.W.

### RESULTS OF TREATMENT IN GERMAN HOSPITALS.

An official report dealing with the results of the treatment of wounded soldiers in German hospitals has been issued by the German Army medical authorities. The report relates to the first nine months of the war, and is, therefore, of a very interesting character. Of each hundred soldiers dismissed from the home hospitals in August 1914, 84.8 were classified as fit for service. For the months of September, October, November, and December 1914, and January, February, March, and April 1915, the percentage of men reported as fit for service varied between 87.3 in November 1914 and 91.2 in April 1915. Taking the report as a whole, the averages are as follows: Fit for service, 88.5; unfit, or on furlough, 9.6; died, 1.9. The Berlin correspondent of the official organ of the American Medical Association draws special attention to the fact that the percentage of fit for service has increased steadily, whereas the mortality percentage has decreased. He also remarks that in the present war the percentage of killed will probably be very much greater than in previous wars, because, judging from reports received, the number of wounds caused by the artillery is much greater than formerly.

### D.S.O. FOR AN AMBULANCE WORKER.

In a supplement to the *London Gazette* which was issued on Wednesday evening it is recorded that the King has conferred the D.S.O. on Captain Stanley Alwyn Smith, of the No. 3 Field Ambulance of the Canadian Army Medical Corps, for conspicuous gallantry and devotion to duty at Festubert on the night of May 20. The official record states that Captain Smith, with a party of eight men, went out voluntarily to remove the wounded from an orchard whilst under heavy fire, and eventually succeeded in bringing all into safety. Four of the eight men of the rescue party were wounded, and two of them have since died. The same issue of the *Gazette* contains a large number of awards by the Tsar to men of the British Army, and we are glad to see that ambulance men and members of the Royal Army Medical Corps figure prominently in the list of recipients. Space does not allow us to give a full list of these awards, but most of them have already been awarded the Distinguished Conduct Medal for brave acts performed under circumstances which have already been described in various issues of The Hospital.

### EXPEDITIOUS RED CROSS SERVICE.

The Kingston Union Farm Colony, by favour of the Guardians and with the consent of the Local Government Board, was handed over to the Kingston and Surbiton Red Cross Committee on July 1. The Red Cross workers of Kingston, Surbiton, and Ditton, men and women, under trained supervision, threw themselves heart and soul into the work of converting for hospital purposes the buildings forming the farm colony. They provided 250 beds, and have introduced into these bungalows an atmosphere of comfort and attractiveness which they never possessed before. The Red Cross Committees have already expended upwards of £1,000 on equipment, and they will further provide for the partial maintenance of the new institution. Much volunteer labour has been forthcoming. Liberal contributions have already been received from the inhabitants of Surbiton and Ditton, and an urgent appeal is now being made for contributions from the large district of Kingston and the thrifty residents in New Malden. Six weeks is a short time in which to achieve so excellent a result as that attained by the enthusiastic help of the voluntary workers interested in the converted buildings of the Kingston Union Farm Colony.

### REPAIRS AND MAINTENANCE.

What constitutes necessary expenditure in a year when urgent appeals are made to institutions and individuals to economise in every direction? At a meeting of the Coventry and Warwickshire Hospital, Mr. Ilett asked for an explanation in connection with the decorations in the out-patient department, for which tenders had been accepted amounting to upwards of £60. Mr. Wormell, the chairman of the meeting, explained that the tenders were for the cleaning of the smaller consulting and casualty rooms in the out-patient department, which were dirty and in bad condition. The medical and surgical staff impressed upon the committee that this was an urgent matter, and there was no alternative but to have the work done, seeing that it had already been delayed too long. The committee had recognised the necessity for economising by delaying to a future time the repainting of the central hall. No doubt, for hygienic reasons alone, the so-called "decorations" of the workrooms surrounding the central hall at the Coventry Hospital had to be done, seeing that no casualty or consulting or examination rooms in any hospital worthy of the name ought ever to be permitted to remain "in a dirty and bad condition." We are confident that no efficient hospital committee will delay repairs and maintenance work which is demanded on hygienic grounds.

### FEVER HOSPITAL HUMOURS.

At Hadley, Shropshire, at a meeting of the Wellington Rural Council on the 19th instant, a lively discussion arose as to the selection of a site by the Joint Committee of the County Council for a Shropshire Joint Isolation Hospital at Hadley. Strong objection was taken to the site selected on the ground that it was situated in the centre of one of the most prosperous villages, Hadley, and to pay a large sum for it when there were, Colonel Patchett declared, other suitable sites free from this objection, which might be had for a nominal sum, was out of all reason. The Rev. W. B.

MacNab, in the course of the discussion, said he had made the owner of the land, Mr. T. Crump, an offer "to brush his boots for six days if the said owner would induce the Local Government Board not to sanction the scheme." Mr. Crump's rejoinder to Mr. MacNab was, "A still tongue makes a wise head." It later appeared that Mr. Crump had entered into an agreement "for the postponement of the consideration of the purchase of the site for a period of six months after the date of the declaration of peace." A Local Government Board inquiry to consider an application for a loan of £800 for the land and other expenses is being held, at which some racy evidence may be expected. We have little doubt that the Local Government Board inspector will secure for Hadley what it deserves.

### LEGATEES AND LEGACIES.

THE trustees of the late Dr. R. W. Bruce, of Arbroath, have informed the directors of the infirmary of a legacy of £500, "to be kept at interest by them till a sufficient sum has accumulated to defray the cost of a resident surgeon." It appeared, from a question asked by Mr. Norman M'Bain, that the late Mr. Patrick Allan Fraser also left a legacy for the payment of a resident surgeon, but Mr. Macdonald remarked, amidst laughter, "There is no money there." Another native of Arbroath, the late Alexander Ruxton, has bequeathed $1,500, to be divided equally between the infirmary and the hospital, for the maintenance of one or more beds in each institution, subject to an appearance being entered by the directors at the Surrogate Court of the County of New York. We hope the expenses entailed by the latter requirement will not absorb too much of the Ruxton legacy. A further difficulty has arisen as to whether "the hospital" was the Convalescent House, a point which the representative will have to settle with the Court of the County of New York before the money is paid over. We venture to state for the guidance of future legators to hospitals that anyone wishing to leave money to hospitals must contribute at least £1,000 if they desire to defray the cost of maintaining a bed in one of the smaller hospitals, and an infinitely larger sum if the bed is to be maintained in a great clinical hospital.

### THE HOMŒOPATHIC HOSPITAL AND THE WOUNDED.

IN a letter written by one of the medical staff of the Homœopathic Hospital there is a suggestion that the hospital is boycotted, so far as the distribution of the wounded is concerned. There are, we are told, twenty-five beds all ready and waiting, but never a wounded soldier comes to occupy one of them. Operating theatres, *x*-rays, pathology departments, private wards, balconies, roof gardens—all are there as in an auctioneer's catalogue; but they remain vacant, ignored, unappreciated. The only explanation proposed is the insincerity of the War Office, which appeals for help and yet neglects the chances which Great Ormond Street so proudly boasts. Whether the position thus described is the outcome of intention or accident we do not pretend to know, but it is just possible that the military medical authorities are not inclined to view with favour an organisation of practitioners who profess themselves to be more virtuous than their fellows. This, in substance, is the essential position of the man who publicly announces himself as member of a particular medical sect with whom alone, such is the suggestion, a knowledge of the truth resides. He is quite free to adopt whatever measures he believes to be good for his patients without the ostentatious display of his personal platform, and without the offensive implication that other men, just as honest and capable as himself, are dwellers in darkness. Doubtless he knows his public and has his reward, but to expect the good will of those whom he traduces is just a little too much.

### THE THREE YEARS' SYSTEM OF TREASURERSHIPS.

THE governors of the Leicester Royal Infirmary are to be congratulated on securing Sir William W. Vincent, J.P., as their treasurer, in succession to Mr. S. Francis Stone. Sir William Vincent has for a large number of years played a prominent part in the public and business life of the borough. He joined the Town Council twenty-five years ago, and was elected Alderman in 1898. He has twice filled the office of Mayor—the second time in the Coronation year of King George V. He is one of the town's representatives and the present chairman of the Derwent Valley Water Board. He received his knighthood in 1912. Sir William Vincent has been a member of the board of the infirmary for many years, and until a few years ago was the chairman of the finance committee. He is thus intimately acquainted with the work and needs of the institution. It is proposed that the present and future treasurers hold office for three years, and the board hope by this means to widen the circle of interest and strengthen the financial position of the institution. Sir William Vincent's standing as a sound financier and administrator should be greatly to the benefit of the institution. Another appointment which is of interest is that of Sir Samuel Faire, J.P., who has been elected by the trustees of the charity of Mr. Benjamin Sutton one of the trustees of that charity in the place of the late Mr. S. F. Stone. The income of the trust funds is applied to deserving and needy in-patients of the infirmary.

### THE SHORTAGE OF CUBE SUGAR.

DURING the last few weeks the shortage of cube sugar has become acute, resulting in the Local Government Board issuing a circular letter to the Boards of Guardians drawing attention to this, and suggesting that the Guardians should take at once into consideration the advisability of discontinuing its use in any cases in which granulated sugar would sufficiently answer the purpose. They point out that there is no material difference between the two, as granulated sugar was practically the raw material from which cube sugar is made. Boards of

Guardians are generally in the habit of purchasing nothing but cube sugar for their infirmaries and other institutions, and if they fell in with the proposal of the Local Government Board they would materially assist the Government in a time of scarcity. Those who partake of the food in the institutions are ignorant, as a rule, of the kind of sugar that is used, so that they would suffer no hardship if granulated sugar was substituted for the manufactured cubes. The advice offered by the Board is quite good, and should be followed in all districts. The available supplies of cube sugar are unevenly distributed, and it may be that the authorities in those localities where sugar in this form is readily obtainable may not be convinced of the necessity for substituting other forms of sugar unless they know that the general shortage of cube sugar is a reality, notwithstanding that in some places little difficulty is experienced in buying it.

### DR. WALKER, OF PETERBOROUGH.

Very hearty congratulations are due to Dr. Thomas James Walker, of Peterborough, who has been made a freeman of the city where he was born eighty years ago, and where he has been in practice for fifty-five years. Dr. Walker represents a type of medical practitioner perhaps less common now than in the past generation. Living away from any of the great medical centres, he has long been widely known as a highly capable and efficient member of his profession; and without professing to be a "specialist," he is recognised as speaking with authority in more than one department of practice. Some two or three years ago he gave the introductory address at the opening of the session at one of the London throat hospitals, and was afterwards entertained to dinner by a very representative gathering. The company included a number of his former house surgeons, some of whom spoke in grateful tones of the training they had received at the hospital at Peterborough. Indeed, to the initiated—and the circle is not a narrow one—a term of service spent with Dr. Walker has often been one of the most valuable recommendations which a young man could boast. Appropriate to the hour, we note that of his nine sons—the company is enriched by four daughters—six hold commissions in H.M. Forces. Our venerable *confrère* has all our respect and regard, and we venture to add an expression of these to his most prized possessions—"Honour, love, obedience, troops of friends."

### THE NEW SECRETARY OF THE STOCKPORT INFIRMARY.

The committee of the Stockport Infirmary has lost no time in appointing a successor to Major Cyril Tyler, who, as announced in our columns recently, has retired from the secretaryship which he has held with marked ability for a long course of years. The advertisement issued by the committee in inviting applications for the position stipulated that whole-time service must be given, and 130 applications were received. The house stewards selected six of the applicants to appear before the full committee on Thursday of last week, and in the result Mr. Hedley Lucas, of Cardiff, was appointed. It is rather curious that Mr. Lucas should come back to the town contiguous to the city in which most of his hospital experience and training thus far have been obtained. He occupied a position as hospital clerk at the Royal Infirmary, Manchester, for two and a-half years, and later was for five years clerk to the superintendent of Monsall Hospital, Manchester. Less than two years ago he was appointed assistant secretary of King Edward VII.'s Hospital, Cardiff. Tribute to Mr. Lucas's work was paid in testimonials from Mr. Leonard D. Rea, secretary and general superintendent, King Edward VII.'s Hospital, Cardiff; Mr. Walter G. Carnt, general superintendent and secretary, Royal Infirmary, Manchester; and Mr. J. L. Paton, the headmaster of the Manchester Grammar School.

### MEASLES AND THE PUBLIC.

There is still a notion widely spread among the public that measles is one of the minor ills of life, and that for the individual it is inevitable. Hence its occurrence is often accepted with complacency, and when once it has appeared in a household little or no attempt is made to protect the younger members from its incidence. Indeed, the conviction is often announced that they had better "get it over." Possibly the name is in some measure responsible for these views and practices. Certainly to the lay mind it has a much less alarming quality than scarlet-fever. Yet the latter is responsible for a much smaller number of deaths, though this is not to say that it has a lower death-rate. In any event, the facts are more than sufficient to justify an attempt to educate the public in the conviction that measles ought to be regarded as a disease involving definite risks, and that to expose children to the infection is a policy wholly unjustifiable. An effort in this direction is being made by the National Association for the Prevention of Infant Mortality. A short and simple statement of the dangers of the disease, of its early symptoms, and of the precautions which must be taken to prevent its spread, particularly among school children, has been prepared, and copies may be had, for a nominal charge, on application to the Secretary, 4 Tavistock Square, W.C. One of the head-lines in the circular runs, "How the Child Should be Treated," but the following paragraphs, very wisely, say nothing about treatment in the ordinary sense of the word. They are confined to directions for preventing the spread of infection, and hence the terms of the reference are not quite happily chosen.

### THE LOUSE AND ITS RELATION TO DISEASE.

A pamphlet bearing this title has just been issued from the Entomology Department of the National History Museum. In some sense it is directed to the spread of information among the public, but its special justification is the liability of our soldiers to suffer from the pests it describes. Loathsome enough in themselves, these creatures carry the risk of even worse consequences in their train. The proposition that typhus fever is conveyed by the clothes-louse, and possibly by the head-louse also, seems to be fairly well established. It has become part of a general doctrine of the truth of which there is indeed convincing proof—namely, that many diseases due to parasites are distributed by the agency of insects. A great deal of what is known as tropical medicine illustrates this truth, and its recognition is one of the most impressive advances of modern medicine. The poison, as of typhus, for example, is communicated to the victim by the bite of the louse, and in this way gains admission to the blood and poisons the tissues. Or, again, if the irritated parts are scratched, abrasions are produced, and if at the same time the parasite is crushed, an infection from it may be conveyed to the body. Hence the recommendation to the victim of these parasites not to scratch the irritated area. The advice is a counsel of perfection, for the bites of these vermin are most irritating. Anyone affected by them should at once take medical advice; they are easily destroyed in civil life, but in war, where numbers of men may be affected, the task is a more difficult one. But to tell the patient not to scratch himself is to forget that "there never was philosopher yet who could endure the toothache patiently."

### A SURGEON'S EXPERIENCE AT THE FRONT.

Mr. Russell Howard, who has lately returned from France, related some interesting experiences in a lecture which he recently delivered at Loughton. After the evacuation of Antwerp the Belgian Red Cross Department was almost necessarily disorganised, and a detachment from England was sent out to try to meet the situation. Major Stedman, who was in command, managed in the course of a few days to organise a hospital of eighty beds in a convent, but the necessary food and supplies were sadly to seek. The time was not one for discussing the rights of property, and the only way to get anything was simply to take it; without this primitive method they would not have had even food for the patients. On the other hand, there arrived to them from England a dozen cases each one containing 250 pairs of socks, very valuable, no doubt, but quite in excess of the situation. At Boulogne, during the retreat from Mons, the confusion was extreme and the Red Cross workers had to steal trucks, engines, and even engine-drivers in order to organise anything like decent care for the wounded. It was impossible for the surgical work to be satisfactory, and on one occasion Mr. Howard had to operate in a room so small that the patient's legs protruded through the window. The medical work in reference to typhoid and to tetanus reached a high level as a result of the inoculation method. In a hospital in the south of France the cook, when the war broke out, was *chef* at the Manhattan Club, New York, at a salary of £800 a year, but he was more than content to work for the wounded at an income of a halfpenny a day. On one occasion Mr. Howard, having no passport, was apprehended as a spy, but was released on production of his golf card, and of the ticket of membership of the Automobile Association. His reminiscences are full of interest, and perhaps he may be persuaded to put them in a permanent form.

### THE L.G.B., THE GUARDIANS, AND ECONOMY.

The Local Government Board has addressed a circular-letter to all local authorities drawing attention to the need of strict economy. The letter has been widely discussed at meetings of Boards of Guardians throughout the country. In many cases it has led to protests and to suggestions that the House of Commons should show an example by cutting down its own expenditure, especially as regards the salaries of Ministers and members and the printing of Blue-books and Reports. On the whole, however, the circular has received the serious consideration it deserves, and many Boards have adopted resolutions postponing or curtailing expenditure upon structural alterations not urgently required, and have ordered changes in dietaries or in administrative details leading to a reduction in the cost of the maintenance of inmates. At Colchester one of the Guardians boldly asserted that the workhouses might be closed. This is, of course, an exaggeration, but there can be no doubt that a very great economy could be effected if Boards of Guardians would combine and pool all their institutions in the combined area. Many workhouses are now only partly full, and in many counties a quarter to a third of the institutions could be closed to-day without any hardship to the inmates. Mr. Williams, the Local Government Board Inspector for Wales, in referring to the proposal to amalgamate the workhouses in Montgomeryshire, said that three workhouses had been closed in Cardiganshire with economic advantage.

### THIS WEEK'S DRUG MARKET.

Business in the drug market continues active, in spite of the fact that we are in the middle of what is at normal times the holiday season. British makers of bismuth preparations, iodides, mercurials, morphine, codeine, etc., are still working at full pressure in their attempt to supply the abnormally large demands for these products. The high values of synthetic drugs are well maintained, and several of them, including aspirin, sulphonal, resorcin, salol, and phenacetin, are again dearer. There is practically no change in the position of cod-liver oil, but the high prices asked continue to keep would-be buyers off the market. Thymol is very scarce, and high prices are asked for the small quantities obtainable. The price of santonin has a lower tendency. Oil of sandalwood is dearer. Cocaine has an upward tendency in price. The values of citric and tartaric acid are not so firmly maintained, and essence of lemon is lower in price.

